# Long Term Dental Work Force Build-Up and DMFT-12 Improvement in the European Region

**DOI:** 10.3389/fphys.2016.00048

**Published:** 2016-02-23

**Authors:** Mihajlo Jakovljevic, Tatjana V. Kanjevac, Marija Lazarevic, Ristic B. Vladimir

**Affiliations:** ^1^Head of Health Economics and Pharmacoeconomics, The Faculty of Medical Sciences, University of KragujevacKragujevac, Serbia; ^2^Vice Dean for Integrated Academic Studies of Dentistry, The Faculty of Medical Sciences, University of KragujevacKragujevac, Serbia; ^3^The Faculty of Medical Sciences, University of KragujevacKragujevac, Serbia; ^4^Department for Orthodontics, The Faculty of Dentistry, University of BelgradeBelgrade, Serbia

**Keywords:** oral health, European region, dentist density, capacity building, DMFT-12, EU, CIS

## Introduction

As Mikiko Hayashi noticed in an amazingly poetic way, dentistry remains in majority of national health systems across the globe: “the Cinderella of health care.” Regardless of undisputed progress of scientific knowledge there is a growing gap in service utilization patterns among the world's rich and poor citizens. The first tend to consume much of a rather cosmetic expensive treatments without essential health added value. At the same time almost three billion of people belonging to the low income households, lack access to basic dental services or do not pay a visit to a dentist for years (Hayashi et al., 2014). Although the issue of affordability is high at stakes in these countries, uneven distribution between rural and urban areas adds to the challenge. Prime example is definitely India whose giant population was served by 117,825 registered dentists out of whom almost 90,000 were concentrated in only four out of thirty Indian federal states (Vundavalli, 2014). Another case is Australia with its huge geographic area and recently reported ratio of almost 40,000% differential between dentist density in the suburbs of core coastline cities and desert Aboriginal communities (Tennant et al., 2013).

Due to international efforts addressing global oral health deficiencies national capacities worldwide have increased sharply over past few decades (Petersen, 2003). Important part of this capacity build-up was grounds laid down by establishment of “WHO Oral Health Country/Area Profile Programme” (or “CAPP”) by the World Health Organization (WHO) back in 1990s. Its cause was the fact that evidence based policy needed reliable and internationally comparable field data. The two main WHO Collaborating Centers whom we own existence and maintenance of these public registries are the Niigata University, Japan and Faculty of Odontology, Malmö, Sweden. The first is in charge of Periodontal Country Profiles and the latter pursues the uneasy task of providing broader Country Oral Health Profiles. Nevertheless other comprehensive sources of evidence on oral health status across regions and nations developed independently. FDI World Dental Federation provides access to the its own Data Hub which consists of fusioned national data sources originating from WHO and World Bank (WB) and Globocan official registries. The European Health for All database (HFA-DB) created and updated by the WHO Office for the European Region and refers to a total of 53 countries located in the European continent. Some of the aforementioned investments allowed for revelation of hidden long term national patterns in oral health care and identification of core weaknesses that might serve as appropriate policy targets in future. So far there is scarcity of published evidence comparing efficiency of all European countries in dental workforce build-up and its relationship to the dental health status of school children in a several decades long time horizon.

## Methodology

Measurements we relied on in this study were averaged over country populations in an observed year. Source used was official release of European Health for All Database (HFA-DB). Targeted 53 countries were all located entirely or partially within the WHO geographical boundaries of the European region. Indicators of health care professional personnel capacity (dentist density and graduate dentist annual outputs) and DMFT-12 (Decayed/Missing/Filled Teeth at 12 year olds) as the core indicator of juvenile oral health were observed. Selected indicators are presented in Tables 1, 2. Total decrease of DMFT-12 and total increase in dentist density per 100,000 population were observed as entire span between the first and the last available value reported to WHO by the national authorities. These historical indicator differentials were used to sort out countries through a top-down approach from most successful to the less efficient ones.

**Table 1:** **Oral health indicator DMFT-12 in the European region according to national values reported to WHO HFA-DB**.

**Countries**	**First available DMFT-12 index**	**Last available DMFT-12 index**	**Time span between two observations (Years)**	**Total DMFT—12 decrease**	**Annual DMFT—12 decrease**
Sweden	7.8^1975^	0.8^2011^	36	7.00	0.194
Norway	8.4 ^1973^	1.6^2006^	33	6.80	0.206
Finland	6.9^1975^	0.7^2009^	34	6.20	0.182
Netherlands	7.3^1974^	1.1^2005^	31	6.20	0.2
Switzerland	6.1^1975^	0.82^2009^	34	5.28	0.155
Slovenia	6.9^1984^	1.8^2000^	16	5.10	0.319
Denmark	5.2^1975^	0.6^2012^	37	4.60	0.124
Italy	5.5^1978^	1.1^2004^	26	4.40	0.169
Ireland	5.4^1970^	1.1^2002^	32	4.30	0.134
United Kingdom	4.7^1973^	0.7^2009^	36	4.00	0.111
Hungary	6.1^1976^	2.4^2008^	32	3.70	0.116
EU members before May 2004	4.74^1980^	1.37^2000^	20	3.37	0.168
Slovakia	5.7 ^1975^	2.4^2006^	31	3.30	0.106
Germany	3.9^1992^	0.7^2009^	17	3.20	0.188
Latvia	6.6^1985^	3.4^2004^	19	3.20	0.168
Portugal	4.6^1975^	1.48^2005^	30	3.12	0.104
Czech Republic	5.7 ^1975^	2.6^2006^	31	3.10	0.1
Luxembourg	3.9^1985^	0.8^2006^	21	3.10	0.148
Croatia	7.6^1986^	4.8^2010^	24	2.80	0.117
Greece	3.8^1975^	1.35^2007^	32	2.45	0.076
France	3.5^1975^	1.2^2006^	31	2.30	0.074
EU	4.1^1985^	1.86^2000^	15	2.24	0.149
Belgium	3.1^1972^	0.9^2010^	38	2.20	0.058
Albania	5.9^1983^	3.8^2007^	24	2.10	0.087
Bosnia and Herzegovina	6.2^1997^	4.2^2004^	7	2.00	0.286
Kyrgyzstan	3.1^1973^	1.1^1993^	20	2.00	0.1
Uzbekistan	2.8^1988^	0.9^2001^	13	1.90	0.146
Estonia	4.1^1988^	2.4^2000^	12	1.70	0.142
European Region	3.81^1985^	2.22^2000^	15	1.59	0.106
Cyprus	2.5 ^1990^	1.3^2010^	20	1.20	0.06
Bulgaria	4.2^1979^	3.1^2008^	29	1.10	0.038
Malta	2.3^1975^	1.4^2004^	29	0.90	0.031
Spain	1.9^1975^	1.1^2010^	35	0.80	0.023
Turkey	2.7^1987^	1.9^2007^	20	0.80	0.04
Israel	2.4^1975^	1.66 ^2002^	27	0.74	0.027
EU members since May 2004	4.22^1985^	3.55^2000^	15	0.67	0.045
Russian Federation	3.5^1975^	2.9^2008^	33	0.60	0.018
Commonwealth of Independent States (CIS)	3.49^1986^	3.46^1990^	4	0.03	0.007
Armenia	2.4^1985^	2.4^1990^	**5**	0.00	0
Georgia	2.4^1985^	2.4^1990^	5	0.00	0
Kazakhstan	2.1^1985^	2.1^1990^	5	0.00	0
Montenegro	3.4^2006^	3.4^2006^	0	0.00	0
San Marino	3.7^1987^	3.7^1990^	3	0.00	0
Tajikistan	1.2^1973^	1.2^1990^	17	0.00	0
Turkmenistan	2.6^1985^	2.6^1990^	5	0.00	0
Lithuania	3.6^1985^	3.7^2005^	20	−0.10	−0.005
Ukraine	2.5^1983^	2.8^2008^	25	−0.30	−0.012
TFYR Macedonia	6.5^1986^	6.9^2007^	21	−0.40	−0.019
Romania	1.7^1975^	2.19^2009^	34	−0.49	−0.014
Republic of Moldova	2.3^1986^	3.5^2008^	22	−1.20	−0.054
Andorra	N/A	N/A	N/A	N/A	N/A
Austria	N/A	1.4^2007^	N/A	N/A	N/A
Azerbaijan	N/A	N/A	N/A	N/A	N/A
Belarus	N/A	2.1^2009^	N/A	N/A	N/A
Iceland	N/A	1.4^2005^	N/A	N/A	N/A
Monaco	N/A	N/A	N/A	N/A	N/A
Poland	N/A	3.2^2010^	N/A	N/A	N/A
Serbia	N/A	N/A	N/A	N/A	N/A

^*^DMFT-12 index—Decayed, missing or filled teeth at age 12.

**Table 2:** **Dentist density and graduate dentist output in the European region according to the national values reported to WHO HFA-DB**.

**Countries**	**Dentists density (PP) per 100 000 (First available/Last available)**	**Time span between two observations (Years)**	**Total increase in Dentist Density (PP) per 100 000**	**Annual increase in Dentist Density (PP) per 100 000**	**Dentists graduated per 100 000 (First/Last available value)**	**Number of Dentists (PP) national level (First/Last available value)**	**Number of dentists graduated in a given year (First/Last available value)**
Portugal	11.0^1980^/76.8^2011^	31	65.8	2.12	0.5^1985^/6.9^2011^	1083^1980^/8108^2011^	50^1985^/723^2011^
Cyprus	35.8^1980^/91.5^2011^	31	55.7	1.80	N/A	182^1980^/783^2011^	N/A
TFYR Macedonia	23.6^1980^/78.6^2011^	31	55	1.77	3.8^1980^/7.6^2010^	446^1980^/1622^2011^	72^1980^/156^2010^
Spain	10.5^1980^/63.0^2011^	31	52.5	1.69	1.0^1991^/3.0^2011^	3946^1980^/29070^2011^	369^1991^/1379^2011^
Greece	79.3^1980^/128.5^2011^	31	49.2	1.59	4.3^1980^/3.2^2007^	7646^1980^/14518^2011^	412^1980^/355^2007^
Luxembourg	36.0^1980^/83.1^2012^	32	47.1	1.47	N/A	131^1980^/441^2012^	N/A
Estonia	46.2^1980^/88.0^2011^	31	41.8	1.35	1.6^1980^/2.3^2011^	682^1980^/1179^2011^	23^1980^/31^2011^
Bulgaria	54.6^1980^/90.9^2011^	31	36.3	1.17	2.6^1985^/4.0^2011^	4839^1980^/6682^2011^	231^1985^/290^2011^
Belarus	17.9^1980^/54.1^2011^	31	36.2	1.17	1.8^1990^/2.6^2011^	1724^1980^/5123^2011^	187^1990^/245^2011^
Austria	21.6^1980^/56.9^2012^	32	35.3	1.10	0.0^1998^/1.6^2010^	1622^1980^/4797^2012^	3^1998^/131^2010^
Croatia	37.4^1980^/71.8^2011^	31	34.4	1.11	3,7^1980^/3.5^2003^	1715^1980^/3162^2011^	169^1980^/156^2003^
Latvia	37.0^1992^/70.7^2011^	19	33.7	1.77	2.5^1980^/1.8^2012^	966^1992^/1456^2011^	62^1980^/37^2012^
Romania	31.7^1999^/62.1^2011^	12	30.4	2.53	1.9^1991^/5.9^2011^	7108^1999^/13324^2011^	446^1991^/1263^2011^
Ireland	30.4^1980^/58.0^2012^	32	27.6	0.86	2.1^1980^/1.5^2011^	1033^1980^/2661^2012^	71^1980^/70^2011^
Lithuania	55.2^1992^/82.1^2011^	19	26.9	1.41	1.5^1985^/4.7^2011^	2044^1992^/2486^2011^	54^1985^/141^2011^
Hungary	26.4^1985^/52.5^2011^	26	26.1	1.00	1.3^1985^/2.8^2011^	2808^1985^/5236^2011^	134^1985^/279^2011^
Czech Republic	45.9^1980^/70.8^2011^	31	24.9	0.80	4,3^1980^/2.9^2011^	4743^1980^/7429^2011^	440^1980^/300^2011^
Ukraine	45.4^2000^/67.6^2012^	12	22.2	1.85	2.8^2000^/4.5^2012^	22372^2000^/30688^2012^	1401^2000^/2045^2012^
Armenia	23.0^2000^/42.7^2012^	12	19.7	1.64	0.7^1980^/20.2^2012^	742^2000^/1290^2012^	22^1980^/610^2012^
EU	48.2^1985^/67.0^2011^	26	18.8	0.72	2.3^1980^/2.7^2011^	N/A	N/A
EU members before May 2004	53.6^1992^/71.3^2011^	19	17.7	0.93	2.3^1985^/2.4^2011^	N/A	N/A
Italy	42.2^1993^/59.3^2011^	18	17.1	0.95	0.7^1985^/2.2^2011^	24000^1993^/35183^2011^	410^1985^/1309^2011^
Germany	65.1^1992^/80.1^2011^	19	15	0.79	3.1^1991^/2.7^2011^	52456^1992^/65502^2011^	2444^1991^/2187^2011^
European Region	28.6^1980^/42.5^2011^	31	13.9	0.45	2.0^1985^/2.3^2011^	N/A	N/A
Republic of Moldova	33.7^1980^/46.9^2012^	32	13.2	0.41	1.8^1980^/3.4^2011^	1353^1980^/1670^2012^	70^1980^/122^2012^
Turkey	15.9^1980^/28.4^2011^	31	12.5	0.40	0.7^1980^/1.3^2011^	7077^1980^/21099^2011^	319^1980^/950^2011^
Andorra	48.4^1995^/60.5^2009^	14	12.1	0.86	2.9^2003^/0.0^2009^	31^1995^/51^2009^	2^2003^/0^2009^
Iceland	73.7^1980^/84.2^2012^	32	10.5	0.33	3.1^1980^/1.9^2010^	168^1980^/270^2012^	7^1980^/6^2010^
Kazakhstan	30.9^1980^/41.2^2012^	32	10.3	0.32	1.8^1990^/2.4^2009^	4623^1980^/6920^2012^	306^1990^/374^2009^
Netherlands	41.0^1995^/50.2^2010^	15	9.2	0.61	3.1^1985^/1.7^2011^	6344^1995^/8345^2010^	453^1985^/278^2011^
Switzerland	45.0^1980^/53.6^2011^	31	8.6	0.28	2.1^1980^/1.4^2011^	2841^1980^/4123^2011^	130^1980^/104^2011^
Albania	24.9^1980^/32.9^2006^	26	8	0.31	1.0^1990^/1.3^2010^	665^1980^/1035^2006^	33^1990^/42^2010^
EU members since May 2004	44.2^1980^/51.4^2011^	31	7.2	0.23	2.3^1980^/3.3^2011^	N/A	N/A
Belgium	63.6^1985^/70.4^2011^	26	6.8	0.26	1.4^1993^/1.3^2011^	6273^1985^/7777^2011^	139^1993^/146^2011^
Commonwealth of Independent States (CIS)	24.8^1980^/30.8^2012^	32	6	0.19	2.0^1990^/2.5^2012^	N/A	N/A
Slovakia	44.1^2000^/50.0^2007^	7	5.9	0.84	2.6^1980^/1.0^2009^	2384^2000^/2697^2007^	130^1980^/53^2009^
United Kingdom	48.3^2007^/53.6^2012^	5	5.3	1.06	N/A	29451^2007^/33653^2012^	N/A
Tajikistan	11.8^1985^/15.9^2011^	26	4.1	0.16	1.0^1990^/0.7^2006^	539^1985^/1244^2011^	55^1990^/43^2006^
Russian Federation	28.5^1990^/32.0^2006^	16	3.5	0.22	2.0^1990^/1.8^2004^	42102^1990^/45628^2006^	2930^1990^/2567^2004^
Uzbekistan	13.8^1980^/17.3^2012^	32	3.5	0.11	1.3^1990^/1.3^2010^	2195^1980^/5151^2012^	255^1990^/377^2010^
Slovenia	59.1^1998^/62.4^2011^	13	3.3	0.25	0.8^1980^/1.9^2011^	1171^1998^/1280^2011^	16^1980^/38^2011^
Norway	82.2^1985^/85.2^2011^	26	3	0.11	2.5^1980^/2.7^2011^	3414^1985^/4218^2011^	101^1980^/135^2011^
Kyrgyzstan	15.3^1980^/17.8^2012^	32	2.5	0.08	1.1^1980^/4.8^2012^	554^1980^/973^2012^	38^1980^/262^2012^
Malta	43.2^2009^/45.3^2012^	3	2.1	0.7	2.8^1990^/1.4^2012^	179^2009^/190^2012^	10^1990^/6^2012^
Bosnia and Herzegovina	19.5^1980^/20.7^2010^	30	1.2	0.04	1.8^1980^/3.4^2010^	799^1980^/797^2010^	74^1980^/132^2010^
Georgia	33.2^1996^/33.6^2012^	16	0.4	0.02	1.0^1990^/7.6^2011^	1534^1996^/1509^2012^	55^1990^/340^2011^
France	65.6^2011^/65.3^2012^	1	−0.3	−0.3	3.4^1980^/1.4^2007^	41507^2011^/41740^2012^	1849^1980^/836^2007^
Azerbaijan	28.9^1980^/26.7^2012^	32	−2.2	−0.07	1.3^1990^/1.2^2012^	1777^1980^/2461^2012^	89^1990^/113^2012^
Turkmenistan	16.7^1998^/11.9^2012^	14	−4.8	−0.34	1.6^1985^/0.3^2012^	788^1998^/614^2012^	51^1985^/14^2012^
Finland	85.2^2000^/78.9^2010^	10	−6.3	−0.63	3.5^1980^/3.3^2012^	4410^2000^/4234^2010^	166^1980^/177^2012^
Sweden	91.2^1995^/84.9^2010^	15	−6.3	−0.42	4.4^1980^/2.3^2010^	8048^1995^/7959^2010^	362^1980^/217^2010^
Denmark	84.6^1992^/77.9^2009^	17	−6.7	−0.39	3.2^1980^/2.5^2011^	4373^1992^/4297^2009^	166^1980^/141^2011^
Israel	85.6^1996^/75.6^2011^	15	−10	−0.66	1.4^1990^/1.2^2011^	4867^1996^/5867^2011^	67^1990^/90^2011^
Serbia	47.0^2003^/34.1^2012^	9	−12.9	−1.43	2.9^2002^/7.6^2011^	3516^2003^/2458^2012^	216^2002^/550^2011^
Monaco	115.4^1980^/102.4^2012^	32	−13	−0.41	0.0^2011^/0.0^2011^	30^1980^/37^2012^	0^2011^/0^2011^
Poland	47.3^1980^/33.8^2011^	31	−13.5	−0.43	2.1^1980^/2.5^2011^	16834^1980^/13033^2011^	740^1980^/958^2011^
Montenegro	26.2^1980^/4.5^2011^	31	−21.7	−0.7	N/A	152^1980^/28^2011^	N/A
San Marino	N/A	N/A	N/A	N/A	N/A	N/A	N/A

^*^Ranking was based on average annual improvement to eliminate bias arising from different reporting periods.

## Results

Combined insight into the national professional capacity data reveals few interesting patterns (Table 1). Over the past three decades, dentist density per 100,000 resident population increased sharply across Europe. The list is topped by mostly Mediterranean countries (Portugal, Cyprus, Spain, Greece), continental high-income economies (Luxemburg and Austria) while the remaining ones among top 10 performers belong to Eastern European formerly planned economies (TFYR Macedonia, Estonia, Bulgaria, Belarus). Surprisingly, the upper half of ranked dental health systems is actually dominated by Eastern European countries out of which some are post-2004 EU members (Croatia, Latvia, Romania, Lithuania, Hungary, Czech Republic) others belonged to the Commonwealth of Independent States (CIS) for the most of post Cold War period (Ukraine, Armenia, Republic of Moldova, Kazakhstan). Few regions in Europe actually recorded fall in professional staff density. This was either the case due to satisfactory health system performance such as the Nordic model applied in Finland, Sweden and Denmark. Other countries with significant negative trend noticed where two Western Balkan countries (Serbia and Montenegro), Poland and CIS members Turkmenistan and Azerbaijan. Among these there are few traditional mature market economies of France, Israel and Monaco.

Observation of European historical evolution on DMFT-12 since the middle of 1970s has shown substantially different landscape (Table 2). The list is topped by Scandinavian countries (Sweden, Norway, Finland and Denmark). All other top ten nations marked by high childhood dental health improvements belong to traditional high income societies (Netherlands, Switzerland, Italy, Ireland, and United Kingdom) or recent ones like Slovenia. The remaining part of upper half of the rank list is dominated by diverse Eastern European countries (Hungary, Slovakia, Latvia, Croatia, Czech Republic, Albania, Bosnia and Herzegovina, Kyrgyzstan, Uzbekistan, Estonia) with quite few OECD members prior to 2000s (Germany, Luxembourg, Greece, France, Belgium, Portugal). Large amount of missing DMFT-12 data in certain years or geographical territories or rather short intervals observed placed significant part of bottom ranked countries into the “no-progress” group (equal values reported at baseline and last point in seven countries) with eight countries with non-applicable DMFT-12 calculation. Five nations confirmed worsening of childhood dental health at age 12 and these were Lithuania, Ukraine, TFYR Macedonia, Romania, Republic of Moldova. Vast majority of the entire aforementioned group of non-classified or poor performing health systems are located within Eastern Europe and the Balkans region.

## Discussion

With regards to core oral health indicators such as the index of serious tooth decay (DMFT) most of broad European Region recorded significant achievements since very concerning dental health landscape of the 1970s. Deep Russian recession of 1990s dragging surrounding nations and transitional health reforms taking place throughout Central and Eastern Europe took their toll (Jakovljevic and Getzen, 2016). Nevertheless since the late 1990s things got substantially better in many of these countries, availability of resources became bigger while management of both in- and outpatient dental services was getting more efficient and cost-effective toward the 2000s (Jakovljevic, 2013). These developments affected both the old public and newly evolving, private dental sector. Over several decades improvements in school children were huge. Such advances mostly assumed decreasing frequency of tooth extractions substituted with fillings as well as longer preservation of natural teeth in adults alongside life span. Although there is an evident converging trend in oral health within the European Union (EU) member states few core weaknesses remain. Some of the most prominent are: inter country and inter regional diversity of dental health status indicators among the elderly, lower affordability of dental care to minority groups and the poor and concerning signs of possible worsening of oral health among the European children since the 2000s (Bourgeois et al., 2003). Blossoming of private dental schools in less regulated markets, oversupply and underemployment of graduate dentists are present but more characteristic of Post-Semashko, Eastern European national systems. Some policy makers across the region suspected falling quality of medical services. This might be partially attributable to the growing competitiveness of the private dental sector in Europe and financial incentives to gain larger profit margins.

Previous literature records suspect direct causal link between DMFT-12 and dentist density. Furthermore there is reliable evidence that pediatric dental health is less linked to the local accessibility of dental practitioners and more dependent upon gross national income level, dental expenditure and expected years of education (Pinilla and González, 2009). Nevertheless availability of these data over long term time horizon allows assessment of independent progress in both issues in Europe. Oversight of significant inter-country differences in dental workforce capacities across the continent to some extent masks huge regional intra-country diversity mostly driven by socioeconomic inequalities (Tchicaya and Lorentz, 2014). Contribution of European Commission's agenda in oral public health is development of strategies aimed at closing major gaps in population dental status within the EU and converging national health policy targets (Widström and Van Den Heuvel, 2005). Recent FDI effort actually reveals hidden patterns of dental work force migration and market incentives affecting service provision and unmet demand (Yamalik et al., 2014). Output of dental graduates follows country size as a general rule (Table 1) and it is dominated by Russian Federation, Germany and other large European countries. Some others like Turkey exhibit so far weaker overall dentist practitioner capacity (21,099 in total in 2011 compared to its large population size). Some of similar time lags of the emerging economies compared to mature ones might be explained by the fact that oral health is frequently neglected policy priority in most national health systems (Kandelman et al., 2000).

## Study limitations

Unique data set exploited for this study consisted of national level records reported to WHO HFA-DB. Unlike many other public health indicators present in major international publicly accessible registries, the best available oral health and dentist density data are presented with wide gaps in both individual countries as well as time periods. These missing data gaps are present in many years or entire regions. Therefore all calculations made here are based upon the best available evidence. Conclusions arising from presented facts are therefore based on differentials between the first and the last available data. In order to improve methodological soundness and applicability we presented individual country advances in community oral health in terms of annual rates and total differentials. Although these calculations might serve as an approximate success ratio they are not fully comparable among countries. This is the case because reported annual national values frequently refer to slightly different time horizons. Nevertheless majority of observed historical data belong to the middle of 1970s or early 1980s while most of the last available data belong to the early 2010s.

## Conclusion

The long term trends observed relate to the period of three to four decades. Such insight points out to the broad changes of the landscape of major challenges in the European dentistry. Obvious successes in liquidating great oral health crisis of the 1970s are reflected in a decent dental status of European school children. Serious efforts to build up dental work force capacities are only partially responsible for that success story (Velickovic et al., 2015). Large part of the improvements is actually attributable to the growing living standards, oral health literacy of general population and policy efforts to improve affordability of dental care to the ordinary citizens (Rančić et al., 2015). Nevertheless major upcoming challenges are population aging associated with extended life expectancy and blossoming of prosperity diseases and increased demand for medical care by the elderly. How the European region will cope with these issues remains unclear (Ogura and Jakovljevic, 2014; Jakovljevic, 2015, 2016; Jakovljevic and Milovanovic, 2015). This study points out to the significant regional differences within the continent (Jakovljevic and Getzen, 2016). Eastern EU members as well as Commonwealth of Independent States members were driving the large part of staff density increase due to their intensive transitional health reforms (Jakovljevic et al., 2015). Regardless of such promising changes these countries will remain substantially more vulnerable to the upcoming challenges compared to the traditional market economies of Western Europe (Jakovljevic et al., 2016a,b).

## Author contributions

MJ and TK designed the research questions and concept of this Opinion article. ML and RV acquired selected published data from the public registry European Health for All Database issued by WHO. All four authors interpreted jointly the findings stated in the article and contributed to the final manuscript in important intellectual content.

### Conflict of interest statement

The authors declare that the research was conducted in the absence of any commercial or financial relationships that could be construed as a potential conflict of interest.
